# Magnetic dynamics and nonreciprocal excitation in uniform hedgehog order in icosahedral 1/1 approximant crystal

**DOI:** 10.1038/s41598-023-41292-1

**Published:** 2023-09-02

**Authors:** Shinji Watanabe

**Affiliations:** Department of Basic Sciences, Kyushu Institute of Technology, Kitakyushu, Fukuoka, 804-8550 Japan

**Keywords:** Magnetic properties and materials, Topological defects

## Abstract

The hedgehog state in the icosahedral quasicrystal (QC) has attracted great interest as the theoretical discovery of topological magnetic texture in aperiodic systems. The revealed magnetic dynamics exhibits nonreciprocal excitation in the vast extent of the reciprocal lattice ${\varvec{q}}$-energy $\omega$ space, whose emergence mechanism remains unresolved. Here, we analyze the dynamical as well as static structure of the hedgehog order in the 1/1 approximant crystal (AC) composed of the cubic lattice with spatial inversion symmetry. We find that the dispersion of the magnetic excitation energy exhibits nonreciprocal feature along the N-P-$\Gamma$ line in the ${\varvec{q}}$ space. The dynamical structure factor exhibits highly structured intensities where high intensities appear in the high-energy branches along the $\Gamma$-H line and the P-$\Gamma$-N line in the ${\varvec{q}}$ space. The nonreciprocity in the 1/1 AC and also in the QC is understood to be ascribed to inversion symmetry breaking by the hedgehog ordering. The sharp contrast on the emergence regime of nonreciprocal magnetic excitation between the QC and the 1/1 AC indicates that the emergence in the vast ${\varvec{q}}$-$\omega$ regime in the QC is attributed to the QC lattice structure.

Quasicrystal (QC) has no periodicity but possesses a unique rotational symmetry forbidden in periodic crystals^1–3^. In periodic crystals, the electric states can be understood on the basis of the Bloch’s theorem grounded on the translational symmetry of the lattice. In contrast, in the QC, the Bloch’s theorem can no longer be applied. This makes understanding of the electronic states and physical properties of the QC far from complete one and hence their clarifications have attracted great interest as the frontier of the condensed matter physics as well as the material science.

One of the important remaining issues has been whether the magnetic long-range order is realized in the QC. In the rare-earth based approximant crystals (AC)s which have the local atomic configuration common to that in the QC with periodicity, the magnetic long-range order was observed experimentally^4^. The 4f electrons at the rare-earth atom are responsible for the magnetism. The antiferromagnetic (AFM) order was observed in the 1/1 AC Cd$_6$R (R=Tb, Y, Pr, Nd, Sm, Gd, Tb, Dy, Ho, Er, Tm, Yb, and Lu)^5–7^, and Au–Al–R (R=Gd and Tb)^8, 9^. The ferromagnetic (FM) order was observed in the 1/1 AC Au-SM-R (SM=Si, Ge, and Al; R=Gd, Tb, Dy, and Ho)^10–13^. Recently, the FM long-range order has been discovered in the QC Au–Ga–Tb and Au–Ga–Gd, which has brought about the breakthrough^14^.

Theoretically, lack of the microscopic theory of the crystalline electric field (CEF) at the rare-earth site in the QC and AC has prevented us from understanding the strongly-correlated electron states, in particular, the properties of the magnetism. Recently, general formulation of the CEF theory in the rare-earth based QC and AC has been developed on the basis of the point charge model^15^. This allows us to reveal that the magnetic anisotropy arising from the CEF at the rare-earth site plays a crucial role in realizing unique magnetic structures^16, 17^. Recent theoretical analyses of the effective model taking into account the magnetic anisotropy in the QC Au–SM–Tb have shown that the uniform FM state is stabilized, which provides a candidate for the magnetic structure observed recently in the QC Au–Ga–Tb^17^. Moreover, interestingly enough, uniform hedgehog state has theoretically been shown to be stabilized in the QC^16^. The hedgehog state is the topological magnetic texture whose magnetic moments located at the 12 vertices of the icosahedron (IC) are directed outward from the IC as shown in Fig. 1A, which is characterized by the topological charge $n=+1$^16^.

Recently, the magnetic dynamics of the uniform hedgehog order in the QC has *for the first time* been studied theoretically^18^. It has been revealed that the nonreciprocal excitation appears in the vast extent of the reciprocal lattice ${\varvec{q}}$-energy $\omega$ space. Namely, the dynamical structure factor has been shown to have different values for the reciprocal lattice vector in the opposite direction, i.e., $S({\varvec{q}},\omega )\ne S(-{\varvec{q}},\omega )$. In this report, to get insight into the nonreciprocal excitation, we analyze the dynamical as well as static structures of the uniform hedgehog order in the 1/1 AC.

It is remarked here that the dynamics of the topological magnetic textures^20^ and the nonreciprocal magnetic excitation^21^ has attracted much attention nowadays in periodic crystals^22^. In non-centrosymmetric systems, the Dzyaloshinskii–Moriya interaction for the magnetic moments is known to cause the nonreciprocal excitations^23–31^. The nonreciprocal magnon was also shown to appear in centrosymmetric systems^32–36^. Recently, the nonreciprocal excitation has been found in the Skyrmion crystal on the centrosymmetric lattice^21^, whose mechanism has not been fully understood. Therefore, the present study contributes to the understanding of the nonreciprocal excitations in the topological magnetic textures on centrosymmetric systems.

**Figure 1:**
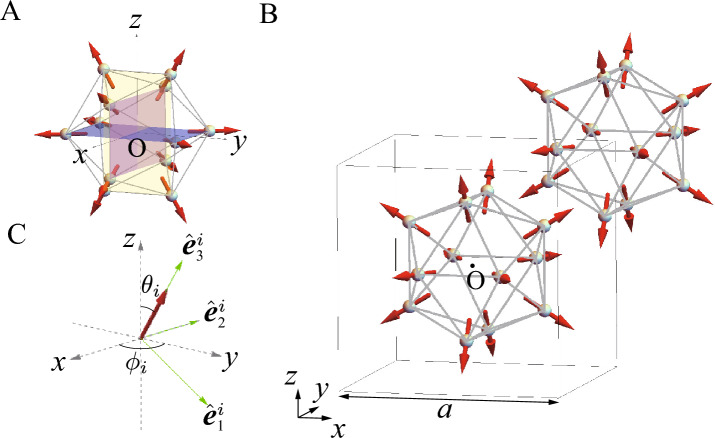
(**A**) The hedgehog state in the IC, where the magnetic moments (red arrows) are directed outward from the IC to the 5-fold axis direction. The rectangle in the IC is colored in purple, pink, and yellow (see text). (**B**) Uniform hedgehog order at the center and corner of the bcc unit cell in the 1/1 AC. The box frame is the cubic unit cell with the side length *a*. (**C**) Local coordinate at the Tb site with the orthogonal unit vectors $\hat{\varvec{e}}_1$, $\hat{\varvec{e}}_2$, and $\hat{\varvec{e}}_3$ where $\hat{\varvec{e}}_3$ is directed to the magnetic easy axis arising from the CEF (see text). (This figure is created by using Adobe Illustrator CS5 Version 15.1.0.).

## Results

### Lattice structure of the IC and 1/1 AC

We consider the lattice structure of the 1/1 AC identified experimentally in the Au$_{70}$Si$_{17}$Tb$_{13}$^13^. The IC is located at the center and the corner of the body-center-cubic (bcc) unit cell with the lattice constant $a=14.726~\mathrm{\text{\AA }}$, as shown in Fig. 1B. The crystal structure is cubic (Space Group No. 204, $Im\bar{3}$, $T_{h}^{5}$), which retains the spatial inversion symmetry.

The hedgehog state in the IC is regarded as the superposition of the coplanar alignment in the three layers orthogonal each other as shown as the purple, pink, and yellow rectangles in Fig. 1A. Each rectangle has the side length $d_{1}a= {0.374} a$ and $d_{2}a= {0.612} a$. As for the direction of the magnetic moments, we consider that each moment is directed to the 5-fold axis (but not the pseudo 5-fold axis on the distorted IC) whose direction is given by the vector passing through each vertex of the regular IC from the center. Namely, the magnetic moment at each vertex of the rectangle in Fig. 1A is given by ${\varvec{S}}_i=S(\pm \frac{1}{\sqrt{\tau +2}},\pm \frac{\tau }{\sqrt{\tau +2}}, 0)$ for the $i=1$, 3, 11, and 12th site (purple rectangle), ${\varvec{S}}_i=S(\pm \frac{\tau }{\sqrt{\tau +2}}, 0, \pm \frac{1}{\sqrt{\tau +2}})$ for the $i=5$, 6, 8, and 9th site (pink rectangle), and ${\varvec{S}}_i=S(0,\pm \frac{1}{\sqrt{\tau +2}}, \pm \frac{\tau }{\sqrt{\tau +2}})$ for the $i=2$, 4, 7, and 10th site (yellow rectangle). Here, *S* is the magnitude of the total angular momentum, which is referred to as “spin” hereafter, and $\tau$ is the golden mean $\tau =(1+\sqrt{5})/2$.

### Minimal model for magnetism in rare earth based QC and AC

We employ the minimal model for the rare earth-based QC and AC introduced in Ref.^19^:

1 $$\begin{aligned} H=\sum _{\langle i,j\rangle }J_{ij}{\varvec{S}}_i\cdot {\varvec{S}}_j-D\sum _{i}({\varvec{S}}_i\cdot \hat{\varvec{e}}^{i}_3)^2, \end{aligned}$$

where ${\varvec{S}}_i$ is the “spin” operator at the *i*th Tb site with $S_i=6$ and $J_{ij}$ is the exchange interaction for the “spins” at the *i*th and *j*th Tb sites. The second term represents the magnetic anisotropy arising from the CEF, where $\hat{\varvec{e}}{^{i}}_3$ is the unit vector along the magnetic easy axis arising from the CEF as shown in Fig. 1C (see Section “Methods” for detail). This model is considered to be effective for broad range of the rare earth-based QC and AC, which is not only restricted to the Tb-based system but also is applied to the other rare-earth-based QC and AC.

The ground-state phase diagram of the model (1) applied to the single IC in the large-*D* limit was determined by the numerical calculations for the ferromagnetic (FM) interaction^17^ and the antiferromagnetic (AF) interaction $J_{ij}$^16^, where $J_1$ is the nearest neighbor (N.N.) interaction [5 bonds for each site with the bond length 0.374*a* (1 bond) and 0.378*a* (4 bonds)] and $J_2$ is the next nearest neighbor (N.N.N.) interaction [5 bonds for each site with the bond length 0.610*a* (4 bonds) and 0.612*a* (1 bond)]. In both cases, the hedgehog state was shown to be realized in the ground state phase diagram of $J_2/J_1$. The model (1) was also applied to the 1/1 AC where the nearest neighbor (N.N.) interaction and the next N.N. (N.N.N.) interaction not only for the intra IC but also for the inter IC was considered in the large-*D* limit^16^. The intra IC interaction is the same as noted above. As for the inter IC interaction, the N.N. interaction is set to be $J_1$ [5 bonds for each site with bond length 0.368*a* (4 bonds) and 0.388*a* (1 bond)] and the N.N.N. interaction is set to be $J_2$ [6 bonds for each site with bond length 0.528*a* (2 bonds) and 0.530*a* (4 bonds)]. It was shown in Ref.^17^ that this minimal model for the large-*D* limit explains the magnetic structures of the FM order (ferrimagnet) observed in the 1/1 AC Au$_{70}$Si$_{17}$Tb$_{13}$^13^ and the AFM order of whirling-moment states in the 1/1 AC Au$_{72}$Al$_{14}$Tb$_{14}$^26^.

It was shown that for $J_2/J_1>2$, the hedgehog state and anti-hedgehog state are realized at the center and corner of the bcc unit cell in the ground state^16, 17^. Within the linear spin-wave theory, we find that the uniform hedgehog order is realized in the 1/1 AC as metastable state although the true ground state is alternated hedgehog order as shown in Refs.^16, 17^. In this report, to get insight into the mechanism of the nonreciprocal magnetic excitation in the uniform hedgehog order found in the QC, we analyze the magnetic excitation in the “uniform” hedgehog state in the 1/1 AC by employing the metastable state (local minimum in the energy landscape) but not the true ground state.

### Static structure factor of magnetism

First, let us analyze the magnetic structure of the uniform hedgehog ground state in the 1/1 AC. The static structure factor of magnetism is defined as

2 $$\begin{aligned} F_{s}({\varvec{q}})=\left\langle \left| \frac{1}{N}\sum _{i}{\varvec{S}}_{i}e^{i{\varvec{q}}\cdot {\varvec{r}}_i}\right| ^2 \right\rangle , \end{aligned}$$

where *N* is the total number of the Tb sites. Let us rewrite the position vector of the Tb site as ${\varvec{r}}_{i}={\varvec{R}}_j+{\varvec{r}}_{0m}$, where ${\varvec{R}}_j$ denotes the position of the center of the IC and ${\varvec{r}}_{0m}$ denotes the position of the *m*th Tb site on the IC. The position vector of the IC ${\varvec{R}}_i$ is expressed as

3 $$\begin{aligned} {\varvec{R}}_i=n_1{\varvec{a}}_1+n_2{\varvec{a}}_2+n_3{\varvec{a}}_3, \end{aligned}$$

where $n_i$ is an integer and ${\varvec{a}}_i$ is the basic translation vector of the bcc lattice ${\varvec{a}}_1=\frac{a}{2}(-1, 1, 1)$, ${\varvec{a}}_2=\frac{a}{2}(1,-1, 1)$, and ${\varvec{a}}_3=\frac{a}{2}(1, 1,-1)$. Here, *a* is the lattice constant of the bcc unit cell (see Fig. 1B). The magnetic structure factor (Eq. 2) is derived to be expressed in the convolution form as

4 $$\begin{aligned} F_{s}({\varvec{q}})=F_{L}({\varvec{q}})S_{\textrm{IC}}({\varvec{q}}), \end{aligned}$$

where the structure factor of the lattice $F_{L}({\varvec{q}})$ and the magnetic structure factor on a single IC $S_{\textrm{IC}}({\varvec{q}})$ are given by

5 $$\begin{aligned} F_{L}({\varvec{q}})= & {} \frac{1}{N_{L}^2}\sum _{j=1}^{N_{L}}\sum _{j'=1}^{N_{L}}e^{i{\varvec{q}}\cdot ({\varvec{R}}_j-{\varvec{R}}_{j'})}, \end{aligned}$$

6 $$\begin{aligned} S_{\textrm{IC}}({\varvec{q}})= & {} \frac{1}{12^2}\sum _{m=1}^{12}\sum _{m'=1}^{12}\langle {\varvec{S}}_{m}\cdot {\varvec{S}}_{m'}\rangle e^{i{\varvec{q}}\cdot ({\varvec{r}}_{0m}-{\varvec{r}}_{0m'})}, \end{aligned}$$

respectively. Here, $N_{L}=N_{1}^3$ is the number of the primitive unit cell which contains the single IC i.e., 12 Tb sites (note that the box frame drawn in Fig. 1B denotes the *expanded* unit cell which contains two ICs, i.e., 24 Tb sites) and $N=12N_{L}$ holds. The wave vector is given by

7 $$\begin{aligned} {\varvec{q}}=\tilde{h}{\varvec{b}}_1+\tilde{k}{\varvec{b}}_2+\tilde{l}{\varvec{b}}_3, \end{aligned}$$

where ${\varvec{b}}_i$ is the basic translation vector in the reciprocal lattice with ${\varvec{b}}_1=\frac{2\pi }{a}(0,1,1)$, ${\varvec{b}}_2=\frac{2\pi }{a}(1,0,1)$, and ${\varvec{b}}_3=\frac{2\pi }{a}(1,1,0)$.

We consider the periodic boundary condition along ${\varvec{a}}_i$ for $i=1,2,$ and 3. Then, $\tilde{h}$, $\tilde{k}$, and $\tilde{l}$ are given by $\tilde{h}=\frac{m_1}{N_1}$, $\tilde{k}=\frac{m_2}{N_1}$, and $\tilde{l}=\frac{m_3}{N_1}$ with integer $m_i$ $(i=1, 2, 3)$. The structure factor of the bcc lattice of the 1/1 AC is calculated as

8 $$\begin{aligned} F_{L}({\varvec{q}})=\frac{1}{N_{L}^2} \frac{\sin ^2(\pi \tilde{h} N_1)}{\sin ^2(\pi \tilde{h})} \frac{\sin ^2(\pi \tilde{k} N_1)}{\sin ^2(\pi \tilde{k})} \frac{\sin ^2(\pi \tilde{l} N_1)}{\sin ^2(\pi \tilde{l})}. \end{aligned}$$

For integer $\tilde{h}, \tilde{k},$ and $\tilde{l}$, the vector ${\varvec{q}}$ in Eq. (7) becomes the reciprocal lattice vector, i.e. ${\varvec{q}}={\varvec{Q}}_{\tilde{h}\tilde{k}\tilde{l}}$, where ${\varvec{Q}}_{\tilde{h}\tilde{k}\tilde{l}}\equiv \tilde{h}{\varvec{b}}_1+\tilde{k}{\varvec{b}}_2+\tilde{l}{\varvec{b}}_3$ with integer $\tilde{h}$, $\tilde{k}$, and $\tilde{l}$. Then, we obtain $F_{L}({\varvec{Q}}_{\tilde{h}\tilde{k}\tilde{l}})=1$. As $\tilde{h}, \tilde{k}$, and $\tilde{l}$ deviate from the integer values, $F_{L}({\varvec{q}})$ decays rapidly in the finite-size system. For the bulk limit, i.e., $N_1\rightarrow \infty$, $F_{L}({\varvec{q}})$ becomes zero for non-integer $\tilde{h}$, $\tilde{k}$, and $\tilde{l}$, which yields $F_{L}({\varvec{q}})=\delta _{{\varvec{q}},{\varvec{Q}}_{\tilde{h}\tilde{k}\tilde{l}}}$.

**Figure 2:**
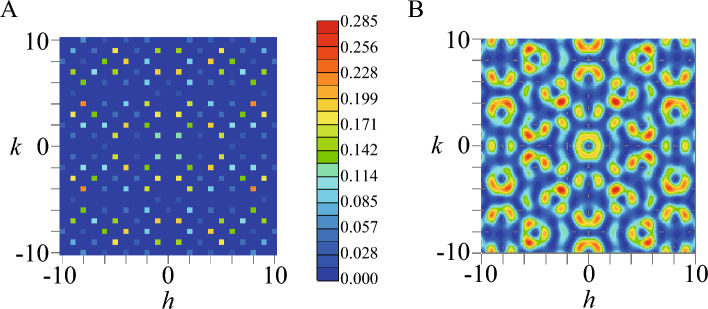
(**A**) The magnetic structure factor of the 1/1 AC $F_{s}({\varvec{q}})$ for ${\varvec{q}}=\frac{2\pi }{a}(h,k,0)$ in the *h*-*k* plane. (**B**) The magnetic structure factor of the IC $S_{\textrm{IC}}({\varvec{q}})$ for ${\varvec{q}}=\frac{2\pi }{a}(h,k,0)$ in the *h*-*k* plane. The color scale is common to that in (**A**). (This figure is created by using Adobe Illustrator CS5 Version 15.1.0.).

Then, it tuns out that the convolution form in Eq. (4) indicates that the ${\varvec{q}}$ vector giving $F_{s}({\varvec{q}})\ne 0$ is restricted to the integer values of $\tilde{h}$, $\tilde{k}$, and $\tilde{l}$ where $F_{L}({\varvec{q}})$ becomes non zero. We have calculated $F_{s}({\varvec{q}})$ in $N_1\rightarrow \infty$ for ${\varvec{q}}=\frac{2\pi }{a}(h,k,l)$ where *h*, *k*, and *l* are defined by $h\equiv \tilde{k}+\tilde{l}$, $k\equiv \tilde{h}+\tilde{l}$, and $l\equiv \tilde{h}+\tilde{k}$, respectively. For $(h,k,l)\in [-10,10]$, the maximum of $F_{s}({\varvec{q}})$ is identified to be 0.215 at $(h,k,l)=(\pm 8,\pm 4,0)$, $(0,\pm 8,\pm 4)$, and $(\pm 4,0,\pm 8)$. We plot $F_{s}({\varvec{q}})$ for ${\varvec{q}}=\frac{2\pi }{a}(h,k,0)$ in the *h*-*k* plane in Fig. 2A. We have also calculated $F_s({\varvec{q}})$ for ${\varvec{q}}=\frac{2\pi }{a}(h,0,l)$ in the *l*-*h* plane at $k=0$ and $F_s({\varvec{q}})$ for ${\varvec{q}}=\frac{2\pi }{a}(0,k,l)$ in the *k*-*l* plane at $h=0$. The results are the same as those in Fig. 2A where *h* and *k* are replaced with *l* and *h* respectively and *k* and *l* respectively. It is noted here that Eq. (4) is also applied to the QC, where $F_{L}({\varvec{q}})$ is the structure factor of the QC lattice.

Here, to get insight into the magnetic structure on the IC, let us analyze $S_{\textrm{IC}}({\varvec{q}})$ in a single IC for general ${\varvec{q}}$. We have searched the maximum in $S_{\textrm{IC}}({\varvec{q}})$ for $h, k, l\in [-10,10]$. Then, the maximum appears at $(h,k,l)=(\pm 2.532, \pm 4.098 ,0)$, $(0,\pm 2.532, \pm 4.098)$, and $(\pm 4.098, 0, \pm 2.532)$. Figure 2B shows $S_{\textrm{IC}}({\varvec{q}})$ in the *h*-*k* plane at $l=0$. We note that the analytic form of $S_{\textrm{IC}}({\varvec{q}})$ for ${\varvec{q}}=\frac{2\pi }{a}(h,k,0)$ can be derived as

9 $$\begin{aligned} S_{\textrm{IC}}({\varvec{q}})= & {} \frac{1}{36}\left( 1+\frac{\tau }{\tau +2}\right) \left\{ 1-\cos (4\pi hd_2)\right\} +\frac{1}{36}\left( 1-\frac{\tau }{\tau +2}\right) \left\{ 1-\cos (4\pi kd_1)\right\} \nonumber \\{} & {} +\frac{1}{36}\left[ 1-\cos (4\pi hd_1)\cos (4\pi kd_2)+\frac{\tau }{\tau +2} \left\{ \cos (4\pi hd_1)-\cos (4\pi kd_2) \right\} \right] \nonumber \\{} & {} +\frac{2}{9} \frac{\tau }{\tau +2} \left\{ \sin (2\pi hd_1)\sin (2\pi hd_2)\cos (2\pi kd_2) +\cos (2\pi hd_1)\sin (2\pi kd_1)\sin (2\pi kd_2) \right\} . \end{aligned}$$

We have also calculated $S_{\textrm{IC}}({\varvec{q}})$ for ${\varvec{q}}=\frac{2\pi }{a}(h,0,l)$ in the *l*-*h* plane at $k=0$ and $S_{\textrm{IC}}({\varvec{q}})$ for ${\varvec{q}}=\frac{2\pi }{a}(0,k,l)$ in the *k*-*l* plane at $h=0$. The results are the same as those where *h* and *k* in Fig. 2B are replaced with *l* and *h* respectively and *k* and *l* respectively. These are understandable from the symmetry of the hedgehog shown in Fig. 1A. Namely, the alignment of the magnetic moments on the IC is invariant under the permutation of the *x*, *y*, and *z* axes so that the same results of $S_{\textrm{IC}}({\varvec{q}})$ are obtained by replacing $(q_x,q_y,q_z)$ with $(q_z,q_x,q_y)$ and also with $(q_y,q_z,q_x)$^18^.

### Dispersion of magnetic excitation from the hedgehog ground state

 To clarify the property of the magnetic excitation in the uniform hedgehog order in the 1/1 AC, we employ the linear spin-wave theory (see Section “Methods”). Namely, we apply the Holstein-Primakoff transformation^37^ to *H*, the “spin” operators are transformed to the boson operators as ${\varvec{S}}_i\cdot \hat{\varvec{e}}_3^i=S-n_i$ with $n_i \equiv a_i^{\dagger }a_i$, $S_i^{-}=a_i^{\dagger }\sqrt{2S-n_i}$, and $S_i^{+}=\sqrt{2S-n_i}a_i$, where $S_i^{+} (S_i^{-})$ is the raising (lowering) “spin” operator and $a_i^{\dagger } (a_i)$ is a creation (annihilation) operator of the boson at the *i*th Tb site. We retain the quadratic terms of the boson operators since the higher order terms are irrelevant at least for the ground state. By taking the primitive unit cell under the periodic boundary condition, we have performed the numerical calculations in the systems with $N_1=8, 64,$ and 256, and show the results in $N_1=256$.

**Figure 3:**
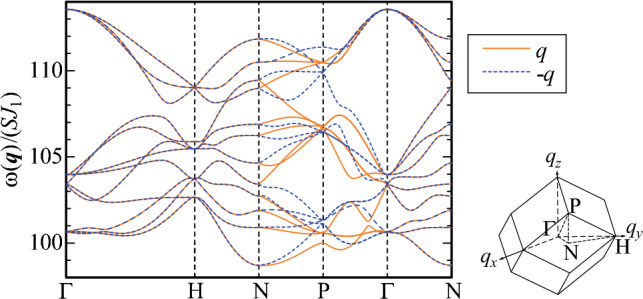
The excitation energy $\omega _{m'}({\varvec{q}})$ (solid line) and $\omega _{m'}(-{\varvec{q}})$ (dashed line) for ${\varvec{q}}$ along the symmetry line in the bcc unit cell illustrated in the inset. The inset shows the unit cell of the bcc lattice of the 1/1 AC in the reciprocal lattice space. (This figure is created by using Adobe Illustrator CS5 Version 15.1.0.).

The excitation energy $\omega _{m'}({\varvec{q}})$ is obtained by diagonalizing the spin-wave Hamiltonian under the assumption that the uniform arrangement of the hedgehog state in the 1/1 AC. The result of $\omega _{m'}({\varvec{q}})$ for ${\varvec{q}}$ (solid line) along the symmetry line illustrated in the inset is shown in Fig. 3. We have confirmed that by assuming the uniform hedgehog ground state, all the excitation energies are positive $\omega _{m'}({\varvec{q}})>0$ for $J_1=1.0$, $J_2=2.3$, and $D=50.0$ as shown in Fig. 3. Positive excitation energies indicate that the assumed ground state is stable at least as a local minimum in the energy landscape. Although the AFM interaction between the ICs conflicts with uniform arrangement of the hedgehog state, the strong uniaxial anisotropy $D=50.0$ as well as the strong N.N.N. AFM interaction $J_2$ inside the IC gives rise to the metastable state within the linear spin-wave theory. It should be noted here that the true ground state is the hedgehog-anti-hedgehog ordered state as shown in Ref.^16^ and the uniform hedgehog order is the metastable state with the higher energy. In this report, the results for $J_1=1.0$, $J_2=2.3$, and $D=50.0$ are presented as the representative case of the uniform hedgehog order. Figure 3 shows $\omega _{m'}({\varvec{q}})$ for ${\varvec{q}}$ (solid line) along the symmetry line illustrated in the inset. Because of the uniaxial anisotropy due to the CEF, the energy dispersion emerges beyond the excitation gap.

We also plot $\omega _{m'}(-{\varvec{q})}$ (dashed line) for ${\varvec{q}}$ along the same symmetry line for the $\omega _{m'}({\varvec{q}})$ in Fig. 3. It is remarkable that clear deviation from the solid line emerges along the N-P-$\Gamma$ line, indicating $\omega _{m'}({\varvec{q}})\ne \omega _{m'}(-{\varvec{q}})$. Namely, nonreciprocal excitation appears in the uniform hedgehog order in the 1/1 AC. We have calculated the magnon dispersion in the collinear FM order in the 1/1 AC and have confirmed that the nonreciprocal dispersion does not appear (Supplementary information, Fig. S1). Since the lattice structure of the 1/1 AC has the inversion symmetry (see Fig. 1B), the emergence of the nonreciprocal dispersion is ascribed to the alignment of the magnetic moments in the hedgehog state (see Fig. 1A). This point will be discussed in detail in the section of analysis of nonreciprocal excitation below.

**Figure 4:**
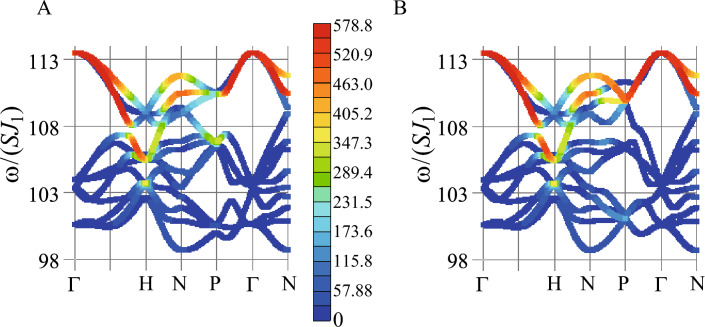
Intensity plots of the dynamical structure factors (**A**) $S_{\perp }({\varvec{q}},\omega )$ and (**B**) $S_{\perp }(-{\varvec{q}},\omega )$ along the symmetry line in the unit cell of the bcc lattice illustrated in the inset of Fig. 3. In (**B**), the color scale is common to that in (**A**). (This figure is created by using Adobe Illustrator CS5 Version 15.1.0.).

**Magnetic dynamical structure factor.** Next, we calculate the magnetic dynamical structure factor defined by

10 $$\begin{aligned} S_{\alpha \beta }({\varvec{q}}, \omega )=-\frac{1}{\pi }\textrm{Im}\langle \textrm{GS}|S_{\varvec{q}\alpha }\frac{1}{\omega +E_{0}-H+i\eta }S_{-{\varvec{q}}\beta }|\textrm{GS}\rangle , \end{aligned}$$

where $|\textrm{GS}\rangle$ is the ground state with the energy $E_{0}$ and $S_{{\varvec{q}}\alpha }$ is the Fourier transformation of the “spin” operator defined by $S_{{\varvec{q}}\alpha }=\frac{1}{\sqrt{N}}\sum _{i=1}^{N}e^{-i{\varvec{q}}\cdot {\varvec{r}}_i}S_{i\alpha }$. We set $\eta =10^{-3}$. The intensity of inelastic neutron scattering is expressed by the dynamical structure factor

11 $$\begin{aligned} S_{\perp }({\varvec{q}},\omega )=\sum _{\alpha ,\beta =x,y,z}(\delta _{\alpha \beta }-\hat{q}_{\alpha }\hat{q}_{\beta })S_{\alpha \beta }({\varvec{q}},\omega ), \end{aligned}$$

where ${\varvec{q}}$ is the incident wavevector and $\hat{q}_{\alpha }\equiv q_{\alpha }/|{\varvec{q}}|$^38^. The results of $S_{\perp }({\varvec{q}},\omega )$ for ${\varvec{q}}$ along the symmetry line in the bcc unit cell illustrated in the inset of Fig. 3 are shown in Fig. 4A. Notable is that the high intensities appear in the high-energy branches, especially along the $\Gamma$-H line and the P-$\Gamma$-N line. In the upper branches, the relatively high and moderate intensities appear.

This is in sharp contrast to the case in the collinear FM order in the 1/1 AC where high intensity in $S_{xx}({\varvec{q}},\omega )$ and $S_{yy}({\varvec{q}},\omega )$ appears continuously from the $\Gamma$ point in the magnon branch with the lowest excitation energy (Supplementary information, Fig. S3). In Fig. 4, the remarkable intensity appears for ${\varvec{q}}$ along the N-P-$\Gamma$ line where the nonreciprocal dispersion $\omega _{m'}({\varvec{q}})\ne \omega _{m'}(-{\varvec{q}})$ appears (see Fig. 3).

In the QC, the dynamical structure factor was calculated in Ref.^18^ where the high intensity appears around the $\Gamma$ point in the high energy region. The high intensities of $S_{\perp }({\varvec{q}},\omega )$ around the $\Gamma$ point, i.e., along the $\Gamma$-H line as well as the P-$\Gamma$-N line shown in Fig. 4A capture this feature. Figure 4B shows the intensity plots of $S_{\perp }(-{\varvec{q}},\omega )$ for the same ${\varvec{q}}$ presented in Fig. 4A. By comparing Fig. 4B with Fig. 4A, we see that the nonreciprocity can be detected as the differences in the intensities of $S_{\perp }(-{\varvec{q}},\omega )$ and $S_{\perp }({\varvec{q}},\omega )$ for ${\varvec{q}}$ along the N-P-$\Gamma$ line. Namely, the nonreciprocal dispersion is remarkable by the intensity differences of the dynamical structure factors in the upper and lower branches.

**Analysis of nonreciprocal excitation.** To analyze the mechanism of the emergence of the nonreciprocal excitation, let us consider how the excitation from the hedgehog ground state propagates in the 1/1 AC.

**Figure 5:**
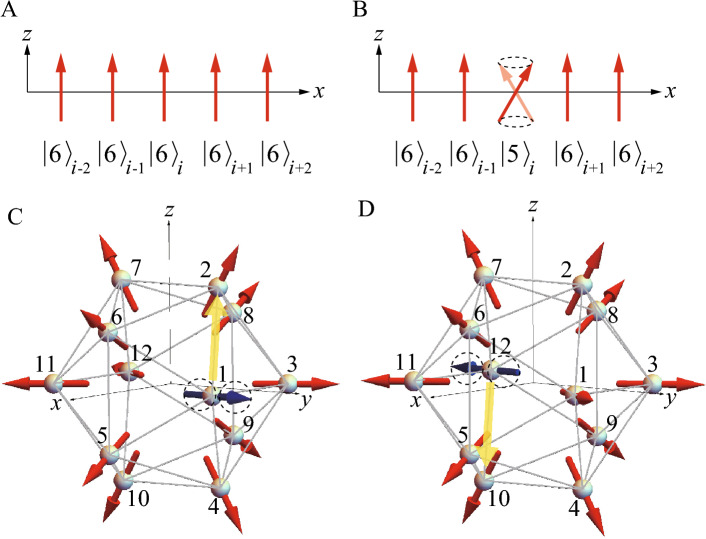
(**A**) The collinear FM order in the *x* chain. Each magnetic moment is aligned along the *z* direction with $J_z=6$. (**B**) The excited state with $J_z=5$ shows precession at the *i*th site. (**C**) The excited state with $J_z=5$ showing precession of the magnetic moment at the 1st site (blue arrow) on the IC. The propagation of the precession to the 2nd site is illustrated by the yellow arrow. (**D**) The excited state with $J_z=5$ showing precession of the magnetic moment at the 12nd site (blue arrow) on the IC. The propagation of the precession to the 10th site is illustrated by the yellow arrow. (This figure is created by using Adobe Illustrator CS5 Version 15.1.0.).

First, for comparison, let us consider the collinear FM ordered state in one dimensional lattice shown in Fig. 5A where the magnetic moment with the total angular momentum $J=6$ is aligned to the *z* direction and the lattice is formed along the transverse direction, which is set as the *x* axis. Namely, $|J=6, J_z=6\rangle$ is realized at each site, which is denoted as $|J_z\rangle _i$, i.e., $|6\rangle _i$ hereafter. Then, let us consider the excited state created at the *i*th site, i.e., $|5\rangle _i$ illustrated in Fig. 5B. This excited state describes the precession around the ordered moment direction, i.e., *z* axis, which propagates to neighboring sites via the exchange interaction $J_1(S_{ix}S_{jx}+S_{iy}S_{jy})$. Namely, the excited state at the *i*th site propagates to the *j*th site as

12 $$\begin{aligned} J_1(S_{ix}S_{jx}+S_{iy}S_{jy})|5\rangle _i|6\rangle _j= & {} \frac{J_1}{2}(S_{i}^{+}S_{j}^{-}+S_{i}^{-}S_{j}^{+})|5\rangle _i|6\rangle _j \nonumber \\= & {} 6J_1|6\rangle _i|5\rangle _j. \end{aligned}$$

Here, the relation of $S_{ix}=(S_{i}^{+}+S_{i}^{-})/2$ and $S_{iy}=(S_{i}^{+}-S_{i}^{-})/(2i)$ and $S_i^{+}|5\rangle =\sqrt{12}|6\rangle$ and $S_i^{-}|6\rangle =\sqrt{12}|5\rangle$ are used. Hence, for the right neighbor site $j=i+1$, the excitation is expressed as the plane wave $6J_1\exp \{i{\varvec{q}}\cdot ({\varvec{r}}_i-{\varvec{r}}_{i+1})\}=6J_1\exp (-iq_{x}a)$ where *a* is the lattice constant. For the left neighbor site $j=i-1$, the excitation is expressed as the plane wave $6J_1\exp \{i{\varvec{q}}\cdot ({\varvec{r}}_i-{\varvec{r}}_{i-1})\}=6J_1\exp (iq_{x}a)$. The superposition of these yields the excitation energy $\varepsilon ({\varvec{q}})$ of the magnon

13 $$\begin{aligned} \varepsilon ({\varvec{q}})=6J_1(e^{iq_{x}a}+e^{-iq_{x}a})=12J_1\cos (q_{x}a). \end{aligned}$$

Since the right moving wave and left moving wave both have the same matrix element $6J_1$ as Eq. (12), the excitation has the reciprocal energy with respect to the transverse wavevector to the ordered moment direction, i.e., $\varepsilon (-q_x)=\varepsilon (q_x)$. It is noted that we have confirmed that the reciprocal magnon dispersion appears in the collinear FM order in the 1/1 AC (Supplementary information, Fig. S2).

Next, let us consider the excitation from the uniform hedgehog ordered state in the 1/1 AC composed of the regular IC. Suppose that among 12 vertices of the regular IC with the $|J=6, J_z=6\rangle$ states, an excited state $|J=6, J_z=5\rangle$ is created at a single site, e.g., the *i*th site. This excited state can propagate to the neighboring sites via the interaction $J_1{\varvec{S}}_i\cdot {\varvec{S}}_j$. Since the $|J,J_z\rangle$ state is defined at each site with the local coordinate, hereafter we denote the state at the *i*th site as $|J_z\rangle _i$. The neighboring $|J_z=5\rangle _i \otimes |J_z=6\rangle _j$ state is exchanged as

14 $$\begin{aligned} J_1{\varvec{S}}_i\cdot {\varvec{S}}_j|5\rangle _{i}|6\rangle _{j} =3J_1\sum _{n=1}^{3}(R_{n1}^{i}-iR_{n2}^{i})(R_{n1}^{j}+iR_{n2}^{j})|6\rangle _i |5\rangle _j \end{aligned}$$

where $R_{nn'}^{i}$ is the rotation matrix at the *i*th site defined in Eq. (20) (see Section “Methods”). Note that as expressed by the $n=3$ component in the right hand side (r.h.s.) of Eq. (14), $S_{i}^{z}S_{j}^{z}$ also contributes to the exchange of $|5\rangle _{i}|6\rangle _{j}$ and $|6\rangle _{i}|5\rangle _{j}$ because of the non-collinear alignment of the magnetic moments. The excitation energy $\varepsilon ({\varvec{q}})$ is obtained by diagonalizing the matrix describing the propagation of $|5\rangle _{i}$ among the $|6\rangle _{j}$ states on all the $j (\ne i)$th sites

15 $$\begin{aligned} H_{i, j}({\varvec{q}})=3J_1\sum _{n=1}^{3}(R_{n1}^{i}-iR_{n2}^{i})(R_{n1}^{j}+iR_{n2}^{j})e^{i{\varvec{q}}\cdot ({\varvec{r}}_i-{\varvec{r}}_j)}, \end{aligned}$$

where *i* and *j* denote the Tb sites inside the primitive unit cell of the bcc lattice.

Let us focus on the excited state at the $i=1$st site $|5\rangle _{1}$ illustrated as the precession of the blue arrow in Fig. 5C. This state propagates to the neighboring $j=2$nd site (see the yellow arrow in Fig. 5C), which is expressed by the matrix element

16 $$\begin{aligned} H_{1,2}({\varvec{q}})=3J_1\frac{1}{\sqrt{\tau +2}}(1+\tau -i\tau )e^{i\{q_x+q_y(\tau -1)-{\tau }q_z \}d}. \end{aligned}$$

Here, ${\varvec{r}}_1=d(1, \tau , 0)$ and ${\varvec{r}}_2=d(0, 1, \tau )$ are used. On the other hand, the matrix element between the spatially inverted sites $i=12$ and $j=10$ (see the yellow arrow in Fig. 5D) is calculated by Eq. (14) as

17 $$\begin{aligned} H_{12,10}({\varvec{q}})=3J_1\frac{1}{\sqrt{\tau +2}}(1+\tau +i\tau )e^{-i\{q_x+q_y(\tau -1)-{\tau }q_z \}d}. \end{aligned}$$

Here, ${\varvec{r}}_{12}=d(-1,-\tau , 0)$ and ${\varvec{r}}_{10}=d(0, -1,-\tau )$ are used. The sign in the $i\tau$ term in Eqs. (16) and (17) are opposite, which signals the nonreciprocal excitation. This can be understood by taking the superposition of Eq. (16) and its spatially inverted term Eq. (17) as done in Eq. (13)

18 $$\begin{aligned} 6J_1\frac{1+\tau }{\sqrt{\tau +2}}\cos [\{q_x+q_y(\tau -1)-{\tau }q_z \}d]+6J_1\frac{\tau }{\sqrt{\tau +2}}\sin [\{q_x+q_y(\tau -1)-{\tau }q_z \}d]. \end{aligned}$$

The $\cos$ term is even function in terms of ${\varvec{q}}$, while the $\sin$ term is odd function giving rise to the nonreciprocal contribution. Namely, the sign change occurs when ${\varvec{q}}$ is replaced with $-{\varvec{q}}$ in the $\sin$ term, which is in sharp contrast to the collinear FM case in Eq. (13). The $\sin$ term also emerges for the other N.N. sites of $j=4, 5$, and 6 to the $i=1$st site and their spatially inverted sites except for the $j=3$rd site (Supplementary information, Section IV).

**Figure 6:**
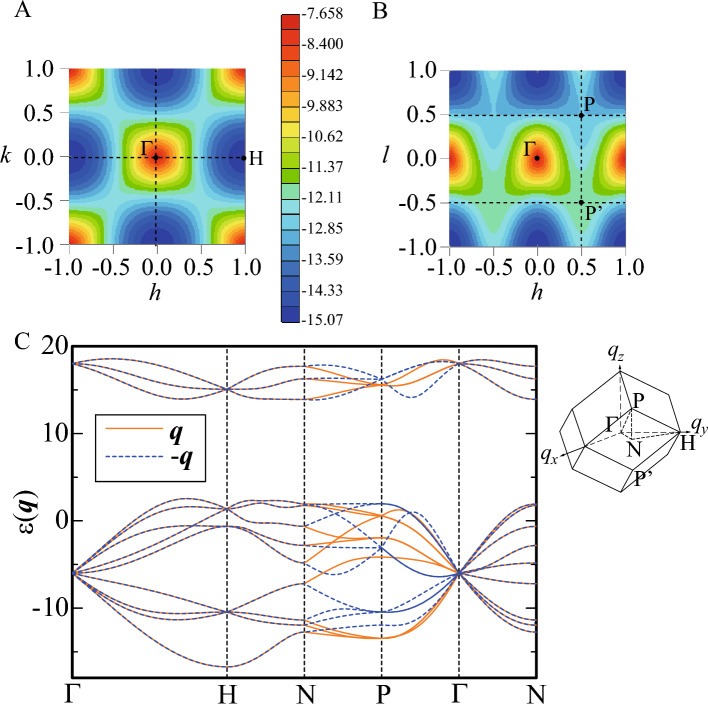
(**A**) $\varepsilon _{1}({\varvec{q}})$ for ${\varvec{q}}=\frac{2\pi }{a}(h,k,0)$ in the *h*-*k* plane. (**B**) $\varepsilon _{1}({\varvec{q}})$ for ${\varvec{q}}=\frac{2\pi }{a}(h,h,l)$ in the *h*-*l* plane. The color scale is the same as that in (**A**). (**C**) $\varepsilon _{m'}({\varvec{q}})$ for ${\varvec{q}}$ along the symmetry line shown in the inset. (This figure is created by using Adobe Illustrator CS5 Version 15.1.0.).

By diagonalizing $H_{i, j}({\varvec{q}})$ in Eq. (15), the excitation energy $\varepsilon _{m'}({\varvec{q}})$ is obtained. Figure 6A shows the lowest excitation energy $\varepsilon _{1}({\varvec{q}})$ in the *h*-*k* plane at $l=0$ for ${\varvec{q}}=\frac{2\pi }{a}(h, k, l)$. The result shows $\varepsilon _1(q_x, q_y, 0)=\varepsilon _1(-q_x, -q_y,0)$ indicating that the reciprocal excitation appears in the $q_x$-$q_y$ plane at $q_z=0$. This explains the result shown in Fig. 3 where $\omega _{m'}({\varvec{q}})=\omega _{m'}(-{\varvec{q}})$ holds along the $\Gamma$-H-N line and the $\Gamma$-N line. We have also calculated $\varepsilon _{1}({\varvec{q}})$ in the *k*-*l* plane at $h=0$ and in the *l*-*h* plane at $k=0$. The result is the same as Fig. 6A with *h* and *k* being replaced with *k* and *l* respectively in the former case and also with *h* and *k* being replaced with *l* and *h* respectively in the latter case. These results show that $\varepsilon _1(q_x, 0, q_z)=\varepsilon _1(-q_x,0,-q_z)$ and $\varepsilon _1(0, q_y, q_z)=\varepsilon _1(0,-q_y,-q_z)$.

Next, we plot $\varepsilon _1({\varvec{q}})$ in the *h*-*l* plane for ${\varvec{q}}=\frac{2\pi }{a}(h,h,l)$ in Fig. 6B. The result shows that $\varepsilon _1(q_x, q_x, q_z)\ne \varepsilon _1(-q_x,-q_x,-q_z)$, namely, nonreciprocal excitation appears, which is most prominent at $(h, k, l)=(1/2, 1/2, 1/2)$. This indicates that the point P and P’ in the reciprocal lattice space (see inset in Fig. 6C) are not equivalent. This explains the result shown in Fig. 3 where the nonreciprocal excitation $\omega _{m'}({\varvec{q}})\ne -\omega _{m'}({\varvec{q}})$ appears along the N-P-$\Gamma$ line.

We show $\varepsilon _{m'}({\varvec{q}})$ for $m'=1, 2, \ldots , 12$ for ${\varvec{q}}$ along the symmetry line displayed in the inset of Fig. 6C. We note that $\varepsilon _{1}(-{\varvec{q}})$ (blue dashed line) at the P point in Fig. 6C is equivalent to the energy at P’ in Fig. 6B. As discussed in Fig. 6B, the nonreciprocal excitation is remarkable along the N-P-$\Gamma$ line not only for the first excited state, i.e., $m'=1$, but also for $m'>1$ as shown in Fig. 6C. These behaviors are consistent with the nonreciprocal feature emerging prominently along the N-P-$\Gamma$ line in Fig. 3. It is noted that to demonstrate the emergence of the analytic form of the sin function [see Eq. (18)], here we discuss the energy dispersion $\varepsilon _{m'}({\varvec{q}})$ for the N.N. interaction $J_1$ in Fig. 6C. Since the energy dispersion $\omega _{m'}({\varvec{q}})$ is plotted in Fig. 3 after subtraction of the ground state energy [see the denominator in r.h.s. of Eq. (10)] for not only the N.N. interaction but also the N.N.N. interaction and uniaxial anisotropy, the energy dispersions in Figs. 3 and 6C are different. However, the nonreciprocal dispersions appear remarkably along the N-P-$\Gamma$ line in both cases.

The origin of the nonreciprocity can be understood as follows: When the magnetic moment at each Tb site located at the position ${\varvec{r}}$ is spatially inverted by operating $-{\varvec{r}}$ with each moment direction being kept (see Fig. 1A), the hedgehog state on the IC is transformed to the anti-hedgehog state. The anti-hedgehog state is defined as the state where the magnetic moment directions are all inverted from those shown in Fig. 1A. The same is applied to the 1/1 AC (see Fig. 1B). Since the magnetic state changes by the space inversion, the inversion symmetry is broken by the hedgehog ordering. Hence, the nonreciprocal dispersion is considered to appear in the excitation from the uniform hedgehog order in the 1/1 AC.

As for the uniform hedgehog order in the QC, the nonreciprocal excitation was reported in Ref.^18^. Since the infinite limit of the unit cell size of a series of ACs (1/1, 2/1, 3/2, $\ldots$ ACs) corresponds to the QC, the infinite folding of the energy dispersion of magnon is expected to occur in the QC. This tendency was indeed confirmed by theoretical calculations in one-dimensional system^39^ and two-dimensional system^40^. This is considered to be reflected in the fine structures of the intensities in the dynamical structure factor for the wide ${\varvec{q}}$-$\omega$ plane in the icosahedral QC as if they are continuum shown in Ref.^18^ where the recursive structure, i.e., the self-similar structure, appears in the intensities. These results suggest that the hedgehog ordering which breaks the spatial inversion symmetry is the origin of the nonreciprocity in the QC^18^.

## Summary and discussion

We have clarified the dynamical as well as static magnetic structure in the uniform hedgehog order in the 1/1 AC. We have shown that the static structure factor of magnetism in the 1/1 AC is generally expressed as the convolution form $F_{s}({\varvec{q}})=F_{L}({\varvec{q}})S_{\textrm{IC}}({\varvec{q}})$ where $F_{L}({\varvec{q}})$ is the lattice structure factor and $S_{\textrm{IC}}({\varvec{q}})$ is the magnetic structure factor of the IC. We have revealed the static structure factor in the uniform hedgehog order by deriving the analytic forms of $F_{L}({\varvec{q}})$ and $S_{\textrm{IC}}({\varvec{q}})$ as well as by calculating them numerically.

We have discovered that the nonreciprocal dispersion of the excitation energy appears prominently along the N-P-$\Gamma$ line in the reciprocal lattice ${\varvec{q}}$ space. The dynamical structure factor of the magnetic excitation in the hedgehog state exhibits the high intensities in the high-energy branches along the $\Gamma$-H line and the P-$\Gamma$-N line. The nonreciprocity $S({\varvec{q}},\omega )\ne S(-{\varvec{q}},\omega )$ is remarkable in the high-energy branches for ${\varvec{q}}$ along the N-P-$\Gamma$ line. The nonreciprocity is clarified to be ascribed to inversion symmetry breaking by the hedgehog ordering whose microscopic source is the strong uniaxial anisotropy *D* as well as the strong N.N.N. AFM interaction $J_2$ inside the IC. In the ferrimagnetic order in the 1/1 AC, the reciprocal dispersion of the magnetic excitation $\omega _{m'}({\varvec{q}})=\omega _{m'}(-{\varvec{q}})$ appears^19^. Since the spatial inversion symmetry is not broken by the ferrimagnetic order in the 1/1 AC, the above statement is consistent with this case. As for the ferrimagnetic order in the QC discussed in Ref.^19^, spatial inversion symmetry is not broken. The intensities of the dynamical structure factors $S({\varvec{q}},\omega )$ and $S(-{\varvec{q}},\omega )$ are almost the same but there exist slight differences in the intensities at some $({\varvec{q}},\omega )$ points^19^ [see $S_{\perp }({\varvec{q}},\omega )$ and $S_{\perp }(-{\varvec{q}},\omega )$ in Fig. S5 in Supplementary Information]. The clarification of this origin is left for future study.

In the hedgehog order in the QC, the nonreciprocal excitation in the hedgehog state appears in the vast extent of the ${\varvec{q}}$-$\omega$ plane where the dynamical structure factor exhibits $S({\varvec{q}},\omega )\ne S(-{\varvec{q}},\omega )$ with considerable intensities^18^. It is noted that we have confirmed on this point for $S_{\perp }({\varvec{q}},\omega )$, i.e., $S_{\perp }({\varvec{q}},\omega )\ne S_{\perp }(-{\varvec{q}},\omega )$. Namely, $|S_{\perp }({\varvec{q}},\omega )-S_{\perp }(-{\varvec{q}},\omega )|$ exhibits considerable intensities (Supplementary Information, Fig. S6B). In the 1/1 AC, the dynamical structure factor shows remarkable difference between the intensities of $S({\varvec{q}},\omega )$ and $S(-{\varvec{q}},\omega )$ for ${\varvec{q}}$ along the N-P-$\Gamma$ line where the nonreciprocal dispersion $\omega _{m'}({\varvec{q}})\ne \omega _{m'}(-{\varvec{q}})$ appears.

These results indicate that the emergence of considerable intensity peaks in $S({\varvec{q}},\omega )$ with different values between ${\varvec{q}}$ and $-{\varvec{q}}$ in the wide ${\varvec{q}}$-$\omega$ plane is attributed to the QC lattice structure.

The present study has pointed out that the inversion symmetry breaking by the uniform hedgehog ordering causes the nonreciprocity $S({\varvec{q}},\omega )\ne S(-{\varvec{q}},\omega )$. There exists a series of the ACs such as 1/1 AC, 2/1 AC, 3/2 AC, $\ldots$, where the $n\rightarrow \infty$ limit in the $F_{n-1}/F_{n-2}$ ACs corresponds to the QC with $F_n$ being the Fibonacci number. Hence, as *n* increases, the size of the unit cell in the $F_{n-1}/F_{n-2}$ AC expands, where the nonreciprocal dispersion of the excitation energy giving rise to $S({\varvec{q}},\omega )\ne S(-{\varvec{q}},\omega )$ is considered to appear when the uniform hedgehog ordering occurs because of the inversion symmetry breaking. Then, in the $n\rightarrow \infty$ limit corresponding to the infinite limit of the unit-cell size of the AC, the nonreciprocity $S({\varvec{q}},\omega )\ne S(-{\varvec{q}},\omega )$ is considered to appear in the uniform hedgehog order in the QC. Hence, the symmetry argument clarified in this study is useful for understanding the nonreciprocal magnetic excitation emerging in the QC.

Thus far, the dynamical structure factor of the icosahedral QC and AC has not been observed experimentally. Hence, the present study is expected to stimulate future experiments to search for unique magnetic states such as the hedgehog and also to observe the dynamical as well as static structure factor of the magnetism.

## Methods

### Linear spin-wave theory for hedgehog state

To analyze the dynamical structure factor for the hedgehog state in the 1/1 AC, the linear spin-wave theory is used in this study. Since the hedgehog state is non-collinear magnetic state, it is convenient to introduce the local orthogonal coordinate at the *i*th Tb site spanned by the unit vectors $\hat{\varvec{e}}^{i}_{\beta }$ $(\beta =1,2$, and 3) (see Fig. 1C) where $\hat{\varvec{e}}^{i}_3$ is directed to the magnetic easy axis of the hedgehog state, i.e., the 5-fold axis direction of the regular IC^16^. The relation between the unit vector $\hat{\varvec{r}}_{\alpha }$ in the global coordinate $(\hat{\varvec{r}}_1\equiv \hat{\varvec{x}}, \hat{\varvec{r}}_2\equiv \hat{\varvec{y}}$, and $\hat{\varvec{r}}_3\equiv \hat{\varvec{z}})$ and $\hat{\varvec{e}}^{i}_{\beta }$ is given by

19 $$\begin{aligned} \hat{\varvec{r}}_{\alpha }=R_{\alpha \beta }^{i}\hat{\varvec{e}}_{\beta }^{i}, \end{aligned}$$

where the direction of $\hat{\varvec{e}}^{i}_3$ is expressed by the polar angles $(\theta _i, \phi _i)$ and $R^{i}$ is the rotation matrix given by^42^,

20 $$\begin{aligned} R^{i}= \begin{bmatrix} \cos \theta _i\cos \phi _i &{}\quad -\sin \phi _i &{}\quad \sin \theta _i\cos \phi _i \\ \cos \theta _i\sin \phi _i &{}\quad \cos \phi _i &{}\quad \sin \theta _i\sin \phi _i \\ -\sin \theta _i &{}\quad 0 &{}\quad \cos \theta _i \end{bmatrix}. \end{aligned}$$

Then, the interaction of “spins” between the *i*th and *j*th sites described by the first term in Eq. (1) is expressed as

21 $$\begin{aligned} \sum _{\langle i,j\rangle }J_{i,j}({\varvec{S}}_i\cdot {\varvec{e}}_{\alpha }^i)({\varvec{S}}_j\cdot {\varvec{e}}_{\beta }^j)\sum _{\gamma }R_{\alpha ,\gamma }^{i}R_{\gamma ,\beta }^{j}. \end{aligned}$$

By employing the relation ${\varvec{S}}_i\cdot \hat{\varvec{e}}_1^i=(S_i^{+}+S_i^{-})/2$ and ${\varvec{S}}_i\cdot \hat{\varvec{e}}_2^i=(S_i^{+}-S_i^{-})/(2i)$ where $S_i^{+}$ and $S_i^{-}$ are raising and lowering “spin” operators, respectively, we apply the Holstein-Primakoff transformation^37^ to *H*. Namely, “spin” operators are transformed to the boson operators as $S_i^{+}=\sqrt{2S-n_i}a_i$, $S_i^{-}=a_i^{\dagger }\sqrt{2S-n_i}$ and ${\varvec{S}}_i\cdot \hat{\varvec{e}}_3^i=S-n_i$ with $n_i\equiv a_i^{\dagger }a_i$ where $a^{\dagger }_i$ $(a_i)$ is the creation (annihilation) operator of the boson at the *i*th site. We retain the quadratic terms with respect to $a_i^{\dagger }$ and $a_i$, which are considered to be at least valid for the ground state. Since the hedgehog state has non-collinear magnetic structure, the anomalous terms such as $a^{\dagger }_{i}a^{\dagger }_{j}$ and $a_{i}a_{j}$ in addition to the normal terms $a_{i}a^{\dagger }_{j}$ and $a^{\dagger }_{i}a_{j}$ appear. The position of the *i*th site is expressed as ${\varvec{r}}_i={\varvec{R}}_j+{\varvec{r}}_{0m}$ where ${\varvec{R}}_j$ denotes the position of the center of the IC and ${\varvec{r}}_{0m}$ denotes the position of the *m*th Tb site on the IC. Hence, by performing the Fourie transformations $a^{\dagger }_{i}=a^{\dagger }_{j,m}=\frac{1}{\sqrt{N_L}}\sum _{\varvec{q}}e^{-i{\varvec{q}}\cdot ({\varvec{R}}_j+{\varvec{r}}_{0m})}a^{\dagger }_{{\varvec{q}},m}$ and $a_{i}=a_{j,m}=\frac{1}{\sqrt{N_L}}\sum _{\varvec{q}}e^{i{\varvec{q}}\cdot ({\varvec{R}}_j+{\varvec{r}}_{0m})}a_{{\varvec{q}},m}$ with ${\varvec{q}}$ being the wave vector, the spin-wave Hamiltonian is expressed by $a^{\dagger }_{{\varvec{q}},m}$ and $a_{{\varvec{q}},m}$. Then, by performing the Bogoliubov transformation, i.e., the para-unitary transformation^41^, the spin-wave Hamiltonian is diagonalized as

22 $$\begin{aligned} H=\sum _{\varvec{q}}\sum _{m'=1}^{12}\omega _{m'}({\varvec{q}})b^{\dagger }_{{\varvec{q}},m'}b_{{\varvec{q}},m'}. \end{aligned}$$

Here, $\omega _{m'}({\varvec{q}})(>0)$ is the energy of the $m'$th spin wave and $b^{\dagger }_{{\varvec{q}},m'}$ $(b_{{\varvec{q}},m'})$ is the creation (annihilation) operator of the boson which is given by the linear combination of the boson operators of $a^{\dagger }_{{\varvec{q}},m}$ and $a_{{\varvec{q}},m}$.

### Supplementary Information

Supplementary Information.

## Data Availability

All the data supporting the findings are available from the corresponding author upon reasonable request.
