# Supplementary material for: Magnetic dynamics and nonreciprocal excitation in uniform hedgehog order in icosahedral 1/1 approximant crystal
Source: Sci Rep. 2023 Sep 2;13:14438. doi: 10.1038/s41598-023-41292-1 (PMC10475090; doi:10.1038/s41598-023-41292-1)
Supplement: Supplementary file 1 — Supplementary Information. [file 41598_2023_41292_MOESM1_ESM.pdf]

Supplementary Information for
" Magnetic dynamics and nonreciprocal excitation in uniform hedgehog order in icosahedral 1/1 approximant crystal "

Shinji Watanabe*

Department of Basic Sciences, Kyushu Institute of Technology, Kitakyushu, Fukuoka 804-8550, Japan

(Dated: August 25, 2023)

I. Magnon dispersion of collinear ferromagnetic order in the 1/1 approximant crystal

In the main text, the excitation energy of the uniform hedgehog order in the 1/1 approximant crystal (AC), i.e., the dispersion is discussed. In this section, we show the magnon dispersion in the collinear ferromagnetic (FM) order whose magnetic moments are directed along the z direction in the 1/1 AC (see Figs. S1A and S1B) for comparison.

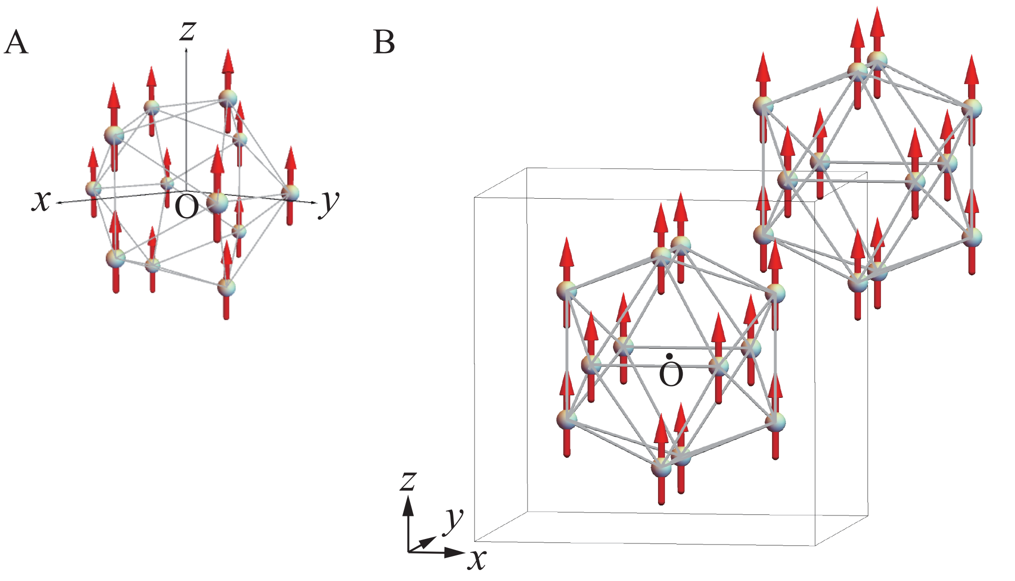

FIG. S1. (A) Collinear FM order on the IC. (B) Collinear FM order in the 1/1 AC.

By applying the linear spin-wave theory [1] to Eq. (1) in the main text, we have calculated the excitation energy $\omega_{m'}(\boldsymbol{q})$ in the collinear FM order in Fig. S1B for $J_1 = -1$, $J_2 = 0$, and $D = 0$. The nearest neighbor interaction J_1 is set not only for intra IC but also for inter IC. The negative J_1 gives the ferromagnetic (FM) interaction, which stabilizes the uniform collinear FM order in the 1/1 AC as the true ground state. The magnitude of the total angular momentum, which is referred to as "spin" S, is set as $S = 6$ as done in the main text. We have performed the numerical calculation of $\omega_{m'}(\boldsymbol{q})$ in the $N = 12N_L$ lattice where N_L is the number of the primitive unit cell $N_L = N_1^3$ with $N_1 = 256$. We plot $\omega_{m'}(\boldsymbol{q})$ (orange line) for $\boldsymbol{q}$ along the symmetry line illustrated in the inset of Fig. S2. The dispersive branch appears from the Γ point which is the ordered vector $\boldsymbol{q} = \boldsymbol{0}$. We also plot $\omega_{m'}(-\boldsymbol{q})$ for the same $\boldsymbol{q}$ (blue dashed line) in Fig. S2. Both the results coincide for all $\boldsymbol{q}$, which indicates that the reciprocal magnon excitation appears.

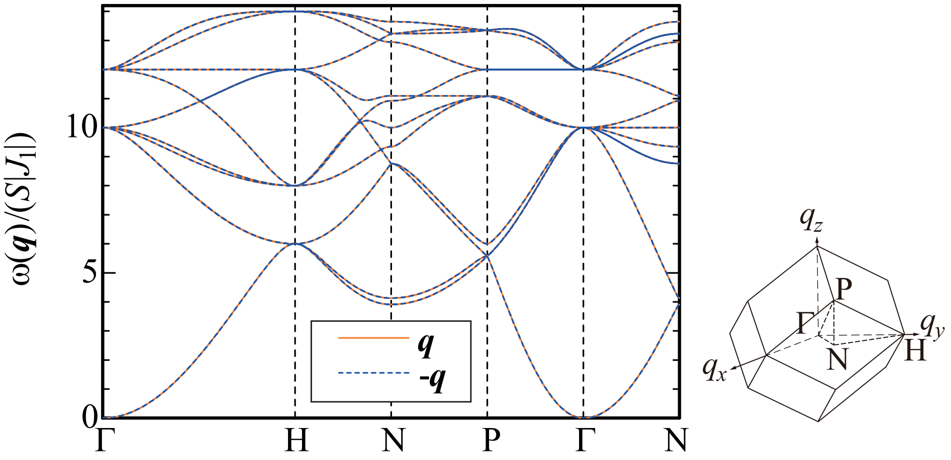

FIG. S2. Magnon energy $\omega_{m'}(\boldsymbol{q})$ (orange line) and $\omega_{m'}(-\boldsymbol{q})$ (blue dashed line) in the collinear FM order in the 1/1 AC for $\boldsymbol{q}$ along the symmetry line shown in the inset. The inset illustrates the unit cell of the body-center-cubic lattice of the 1/1 AC in the reciprocal lattice space.

This can be understood from the viewpoint of the symmetry operation as follows: When the magnetic moment at each Tb site located at the position $\boldsymbol{r}$ is spatially inverted by operating $-\boldsymbol{r}$ with each moment direction being kept (see Fig. S2A), the collinear FM state where all the magnetic moments are directed along the z direction does not change. Since the magnetic state is unchanged by space inversion, the inversion symmetry retains by the FM ordering. This is in sharp contrast to the case of the hedgehog order discussed in the main text.

II. Dynamical structure factor of collinear FM order in the 1/1 AC

We have calculated the dynamical structure factors $S_{\alpha\alpha}(\boldsymbol{q},\omega)$ ($\alpha = x, y$, and z) for $J_1 = -1$, $J_2 = 0$, and $D = 0$. in the collinear FM state by the linear spin-wave theory. The results of $S_{xx}(\boldsymbol{q},\omega)$, $S_{yy}(\boldsymbol{q},\omega)$, and $S_{zz}(\boldsymbol{q},\omega)$ calculated in $N_1 = 256$ are shown in Figs. S3A, S3B, and S3C, respectively. In $S_{xx}(\boldsymbol{q},\omega)$, the high intensity appears in the lowest excitation branch along the Γ-H line and P-Γ-N line. The moderate intensities appear in the lowest and second-lowest branches along the H-N-P line.

The same behavior appears in $S_{yy}(\boldsymbol{q},\omega)$ as shown in Fig. S3B. This is understandable from Fig. S1B. Since the magnon is the transverse wave of the precession of the magnetic moment around the z axis, the magnon dispersion appears equivalently in the x and y directions with respect to the ordered moment (z) direction.

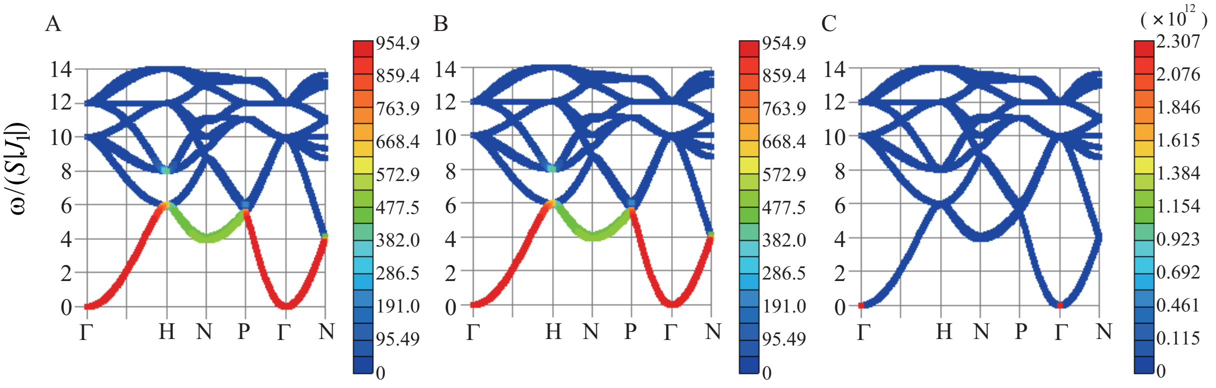

FIG. S3. Dynamical structure factors (A) $S_{xx}(\boldsymbol{q}, \omega)$, (B) $S_{yy}(\boldsymbol{q}, \omega)$, and (C) $S_{zz}(\boldsymbol{q}, \omega)$ for $\boldsymbol{q}$ along the symmetry line illustrated in the inset of Fig. S2.

In sharp contrast, in $S_{zz}(\boldsymbol{q}, \omega)$ the highest intensity colored in red appears at the ground state $\omega = 0$ at the Γ point, i.e., the ordered vector $\boldsymbol{q} = \boldsymbol{0}$, as shown in Fig. S3C. This is typical behavior of the longitudinal component of the dynamical structure factor in the linear spin-wave theory which describes the precession around the ordered moment propagating as the transverse wave.

We have also calculated $S_{xx}(-\boldsymbol{q}, \omega)$, $S_{yy}(-\boldsymbol{q}, \omega)$, and $S_{zz}(-\boldsymbol{q}, \omega)$ for the same $\boldsymbol{q}$ in Fig. S3 and have confirmed that the intensities are the same as $S_{xx}(\boldsymbol{q}, \omega)$, $S_{yy}(\boldsymbol{q}, \omega)$, and $S_{zz}(\boldsymbol{q}, \omega)$, respectively. Hence, in the collinear FM order, the reciprocal magnon is realized in terms of the dispersion and the intensity of the dynamical structure factor.

III. Gd- and Eu-based quasicrystal and AC

In the Gd^{3+} and Eu^{2+} ions with $4f^7$ configuration, the ground multiplet is $^8S_{7/2}$ where the total angular momentum is $J = 7/2$ with the total spin $S = J$ and the total orbital angular momentum $L = 0$. Namely, in the Gd- and Eu-based quasicrystal and AC, the orbital degree of freedom of 4f electrons at the rare-earth site is quenched. Hence, the uniaxial anisotropy arising from the crystalline electric field (CEF) is expected to be absent. Hence, the argument in sections I. and II. is expected to be relevant to the Gd- and Eu (Eu^{2+} case)-based AC.

IV. Analysis of excitation in the hedgehog order in the 1/1 AC

In the main text, the excitation energy in the uniform hedgehog order in the 1/1 AC is analyzed. The excited state $|J_z = 5\rangle_i$ with the precession around the magnetic easy axis i.e.,

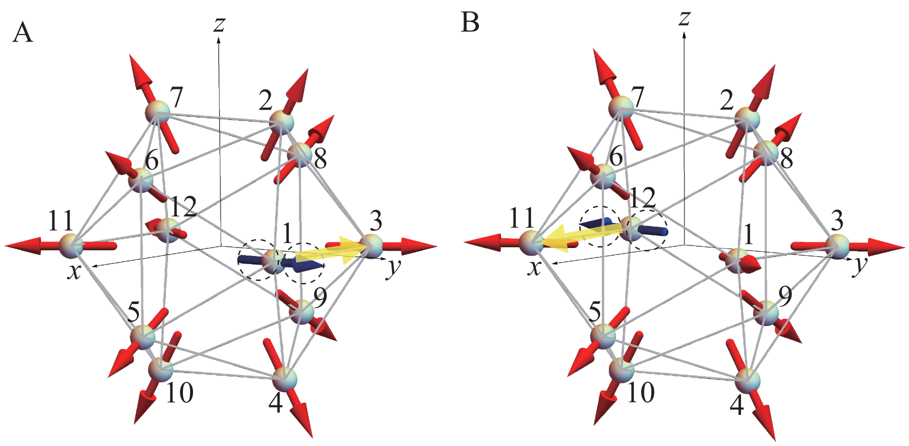

FIG. S4. (A) The excited state with $J_z = 5$ showing precession of the magnetic moment at the 1st site (blue arrow) on the IC. The propagation of the precession to the 3rd site is illustrated by the yellow arrow. (B) The excited state with $J_z = 5$ showing precession of the magnetic moment at the 12nd site (blue arrow) on the IC. The propagation of the precession to the 11th site is illustrated by the yellow arrow.

5 fold axis propagates to the neighboring sites. For example, the matrix element between the excited state at the i = 1st site $|J_z = 5\rangle_1$ and the fully polarized state at the i = 3rd site $|J_z = 6\rangle_3$ (see Fig. S4A) is given by

$$H_{1,3}(\boldsymbol{q}) = 6J_1 \left(1 - \frac{2}{\tau + 2}\right) e^{2iq_x d}. \tag{1}$$

The matrix element for the spatially inverted sites $(i, j) = (12, 11)$ (see Fig. S4B) is given by

$$H_{12,11}(\boldsymbol{q}) = 6J_1 \left(1 - \frac{2}{\tau + 2}\right) e^{-2iq_x d}. \tag{2}$$

The superposition of Eqs. (1) and (2) gives

$$12J_1 \left(1 - \frac{2}{\tau + 2}\right) \cos(2q_x d), \tag{3}$$

which has no sin term. Similarly, the matrix element between the sites located parallel to the y or z axis, i.e., $(i, j) = (2, 7)$ and $(5, 6)$ and that for their spatially inverted sites in Fig. S4 gives $12J_1\{1 - 2/(\tau + 2)\}\cos(2q_y d)$ and $12J_1\{1 - 2/(\tau + 2)\}\cos(2q_z d)$, respectively, which has no sin term.

V. Dynamical structure factor for ferrimagnetic order in quasicrystal

In Ref. 19 in the main text [2], the dynamical structure factor for the ferrimagnetic order in the QC shown in Fig. S5A was discussed on the basis of the model (1) in the main text. In Figs. 6a and 6b in Ref. [2], $S_{yy}(\boldsymbol{q},\omega)$ and $S_{yy}(-\boldsymbol{q},\omega)$ are displayed respectively. In the main text, the dynamical structure factor $S_{\perp}(\boldsymbol{q},\omega)$ for the inelastic neutron scattering is defined by Eq. (11). Here, we show $S_{\perp}(\boldsymbol{q},\omega)$ and $S_{\perp}(-\boldsymbol{q},\omega)$ for the ferrimagnetic order in the QC in Figs. S5B and S5C, respectively, where $\boldsymbol{q}$ is plotted along the symmetry line in the body-center-cubic lattice of the 1/1 AC illustrated in the inset of Fig. S5B. Here, the lattice structure of the QC shown in Fig. S6A is the same as those discussed in Ref. [2] and the parameter $J_1 = -1.0$, $J_2 = -1.0$, and $D = 20.0$ is set for the model Eq. (1) applied to the QC. Similarly to Fig. 6 in Ref. [2], $S_{\perp}(\boldsymbol{q},\omega)$ and $S_{\perp}(-\boldsymbol{q},\omega)$ have almost similar intensities but there exist slight differences in the intensities between $\boldsymbol{q}$ and $-\boldsymbol{q}$ (e.g., near the H point at $\omega/(|J_1|S) \sim 43$).

VI. Dynamical structure factor for hedgehog order in quasicrystal

In Ref. 18 in the main text [3], the nonreciprocity in the dynamical structure factor for the hedgehog order in the QC (see Fig. S6A) was discussed on the basis of the model (1) in the main text. In Fig. 5 in Ref. [3], $|S_{xx}(\boldsymbol{q},\omega) - S_{xx}(-\boldsymbol{q},\omega)|$ was shown to exhibit considerable intensities in the vast extent of the $\boldsymbol{q}$-ω plane. In the main text, the dynamical structure factor $S_{\perp}(\boldsymbol{q},\omega)$ for the inelastic neutron scattering is defined by Eq. (11). Here, we show $|S_{\perp}(\boldsymbol{q},\omega) - S_{\perp}(-\boldsymbol{q},\omega)|$ for the hedgehog order in the QC in Fig. S6B, where $\boldsymbol{q}$ is plotted along the symmetry line in the body-center-cubic lattice of the 1/1 AC illustrated in the inset of Fig. S6B. Here, the lattice structure of the QC shown in Fig. S6A is the same as those discussed in Ref. [3] and the parameter $J_1 = 1.0$, $J_2 = 2.3$, and $D = 50.0$ is set for the model Eq. (1) applied to the QC. Similarly to Fig. 5 in Ref. [3], considerable intensities appear in the wide $\boldsymbol{q}$-ω region, which indicates the nonreciprocal excitations in the hedgehog order in the QC.

[1] Holstein, T. & Primakoff, H., Field dependence of the intrinsic domain magnetization of a ferromagnet, *Phys. Rev.* **58**, 1098 (1940).

[2] Watanabe, S., Magnetic dynamics of ferromagnetic long range order in icosahedral quasicrystal, *Sci. Rep.* **12**, 10792 (2022).

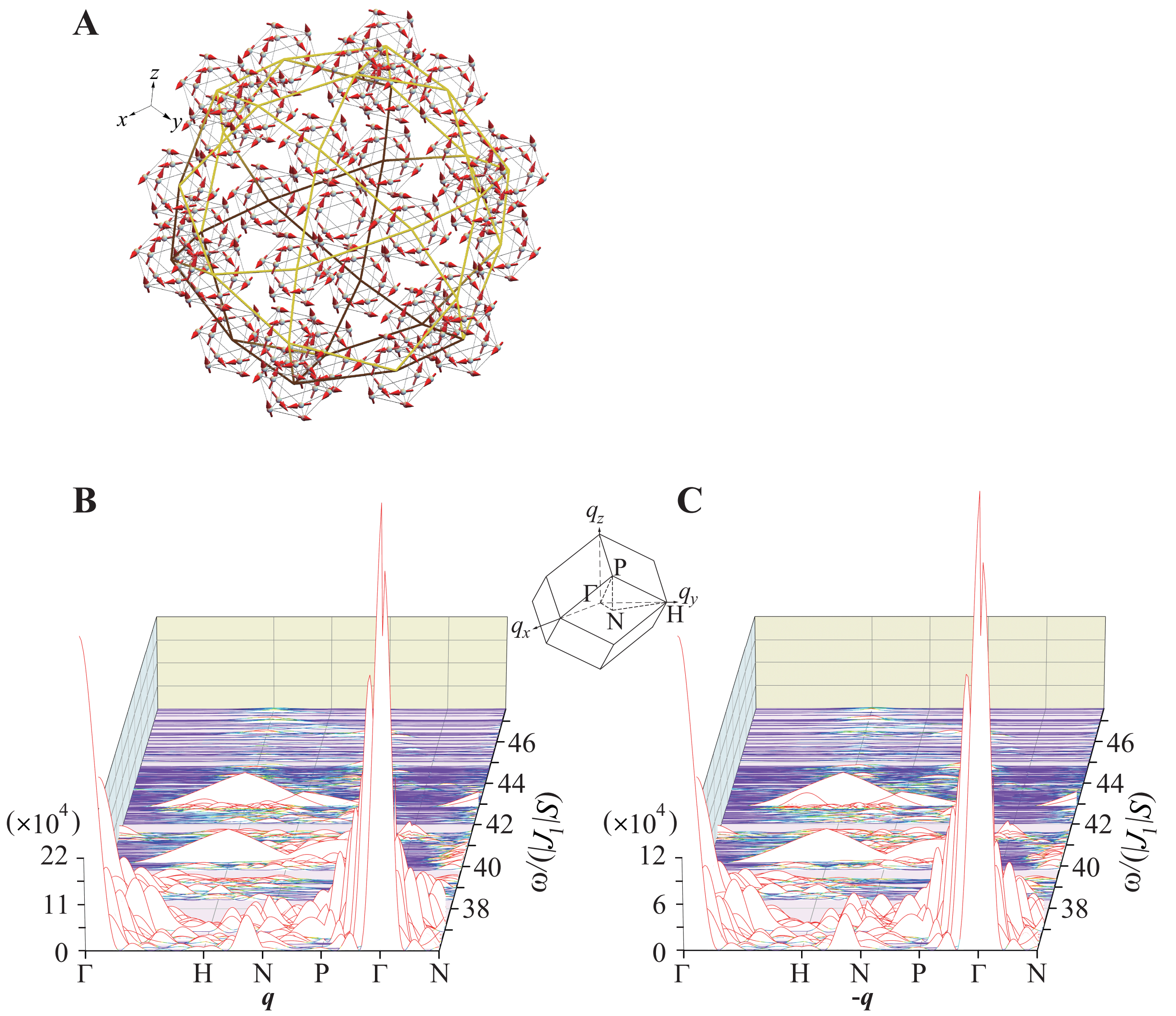

FIG. S5. (A) The uniform ferrimagnetic state in $Cd_{5.7}Yb$-type QC. Green (brown) lines at the front (back) connect the vertices of the icosidodecahedron [2]. (B) $S_{\perp}(\boldsymbol{q}, \omega)$ and (C) $S_{\perp}(-\boldsymbol{q}, \omega)$ for the ferrimagnetic order in the QC, where $\boldsymbol{q}$ is plotted along the symmetry line in the body-center-cubic lattice of the 1/1 AC illustrated in the inset.

[3] Watanabe, S., Magnetic dynamics of hedgehog in icosahedral quasicrystal, *Sci. Rep.* **12**, 15514 (2022).

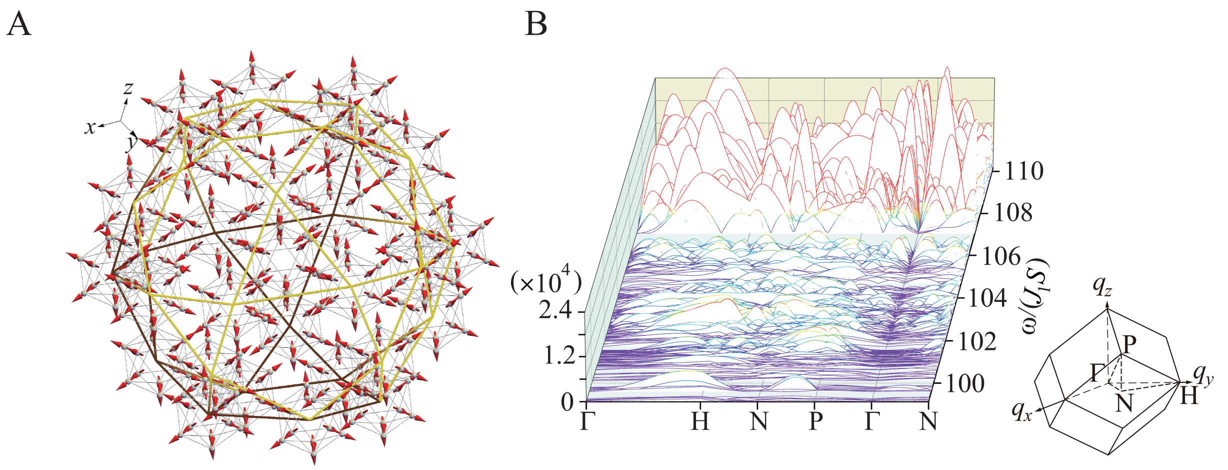

FIG. S6. (A) The uniform hedgehog state in $Cd_{5.7}Yb$-type QC. Green (brown) lines at the front (back) connect the vertices of the icosidodecahedron [3]. (B) $|S_{\perp}(\boldsymbol{q},\omega) - S_{\perp}(-\boldsymbol{q},\omega)|$ for the hedgehog order in the QC, where $\boldsymbol{q}$ is plotted along the symmetry line in the body-center-cubic lattice of the 1/1 AC illustrated in the inset.
